# Novel vs. modified platelet-rich plasma therapy for hair loss treatment: a systematic review and meta-analysis

**DOI:** 10.1097/MS9.0000000000000396

**Published:** 2023-05-19

**Authors:** Kaneez Fatima, Haziq Ovais, Zainab Anwar, Maaz Abdul Latif Motan, Maaz Masood Khan, Aly H. Khowaja, Altamash Shahriyar Ghazanfar, Muhammad A. Khalid, Ayesha Jamil, Yahya Ismail Mujtaba, Sahil Kumar, Ahmed M. Rashid

**Affiliations:** a Department of Medicine, Dow University of Health Sciences; b Department of Medicine, Jinnah Sindh Medical University; c Department of Medicine, Aga Khan University Hospital; d Department of Medicine, Ziauddin University Hospital; e Department of Medicine, Jinnah Medical and Dental College, Karachi, Pakistan

**Keywords:** androgenic alopecia, hair loss, minoxidil, platelet-rich plasma, PRP

## Abstract

Platelet-rich plasma (PRP) therapy, is a stand-alone therapy or is used in combination with other treatments for the treatment of androgenic alopecia. The purpose of this meta-analysis was to compare the improvement in hair density seen, when PRP was used alone and when used as an adjunct.

**Methods:**

Conforming to systematic review and meta-analysis recommendations, we performed a meta-analysis of relevant articles in multiple databases, from inception till May 2022. Randomized clinical trials were included that evaluated the use of PRP alone or used PRP as an adjuvant with previously used therapies. Hair density data at the start of treatment and follow-up after 3 and 6 months was used for analysis.

**Results:**

A review of 255 articles included nine studies, for a cumulative data set of 230 individuals. In comparison to the placebo, stand-alone PRP therapy resulted in a significant increase in hair density (MD=25.39, *P*<0.00001.) PRP combination therapy also showed marked improvement in hair density compared to placebo (MD=34.38, *P*=0.002.) When comparing stand-alone PRP to combination therapy, MD=36.16, and MD=34.63 was observed for the two groups, respectively.

**Conclusions:**

The results of this meta-analysis reaffirm previous studies that suggest the role of PRP in improving hair density in AGA; however, the results cannot justify the use of PRP-combined therapy. Stand-alone PRP therapy should be considered in the management protocols for both men and women, whereas more studies and may be, different combination therapies are required before combined therapy can be included in the management guidelines for AGA.

## Introduction

Highlights

- This systematic review and meta-analysis evaluated the effects of platelet-rich plasma (PRP) when used as monotherapy or in combination with the previously used modalities to treat androgenic alopecia.
- We found that PRP when used alone or in combination shows a significant increase in hair density.
- More studies are required to assess the effects of PRP on other outcomes.

Androgenic alopecia (AGA), described as a nonscarring pattern of hair loss, is a chronic disorder affecting 80% of men and 50% of women^1,2^. Studies conducted have suggested the cause to be a combination of genetic predisposition and the effect of androgen on hair follicles^3^. It is a developing disease that is characterized by inflammation and shortening of the hair and eventually they appear villus-like^4,5^. Hair is an important aspect of self-image and a measure of societal attractiveness. Therefore, suffering from AGA can have detrimental effects on a person’s mental health and can significantly deteriorate the quality of life^6,7^.

Platelet-rich plasma (PRP) therapy has been identified as the treatment option for AGA and has shown significant improvement in hair regrowth at 3 and 6 months when treated^8^. Recently a combination of PRP therapy with mainly topical application of minoxidil and oral finasteride has been introduced^9,10^. Less commonly polydeoxyribonucleotide, dalteparin, and protamine microparticles are also used with PRP^11,12^. PRP can also be combined with other treatment methods and in the early phases of alopecia, combined PRP and a stromal vascular fraction have yielded promising results^13^. Micro-needling and low-level laser therapy can also be used in conjunction with PRP and drugs to treat hair loss^14,15^.

A recent meta-analysis conducted on PRP therapy alone has shown an increase in hair thickness and density^16^. However, to the best of our knowledge, a meta-analysis on PRP therapy and its combination with drugs or other interventions has not been conducted. Hence, the efficacy of PRP alone, and in conjunction with topical and oral drugs, will be compared statistically revealing the best treatment option for AGA hypothetically.

## Methodology

This study was conducted according to ‘Preferred Reporting Items for Systematic Reviews and Meta-Analyses (PRISMA)’ guidelines^17,18^. The study was then evaluated with the AMSTAR 2 critical appraisal tool for systematic reviews that included randomized or nonrandomized healthcare intervention studies^19^.

### Search strategy and study selection

Two independent reviewers, H.O. and Z.A. performed literature searches of all published articles from inception till May 2022 using the databases PubMed, EMBASE, MEDLINE, the Cochrane Database of Systematic Reviews (CDSR), MedlinePlus, Scopus, and Ovid. The authors also searched for databases of gray/unpublished literature and bibliographies of selected articles, registries of ongoing randomized clinical trials, and recently published editorials and reviews related to PRP. The articles were screened in the title, abstract, and full-text and duplicates were removed. The search was done using the Boolean operators ‘OR’ and ‘AND’, and key terms were used such as ‘alopecia’, ‘baldness’, ‘androgenic alopecia’, ‘male pattern baldness’, ‘autologous conditioned plasma’, and ‘platelet-rich plasma’. To collect all published clinical trials, both randomized and nonrandomized articles were included. The articles that fell under the inclusion criteria were those that were published in English, used PRP to treat AGA with saline/placebo in a control group, and had a minimum follow-up of 1 month. The excluded articles were those with less than ten participants, used animals as the experimental group, or had not reported the demographic and baseline characteristics of the patients, and were observational reviews, and case studies. Only randomized control trials (RCTs) were included in the study. Prospective studies or retrospective studies were not included because there were insufficient studies that fulfilled the inclusion and exclusion criteria.

### Data extraction and quality assessment

Data extraction was done by the first investigator (H.O.) and then the second investigator (M.M.K.) rechecked the entire data for accuracy. Disagreements during the extraction process were resolved through consensus. Additionally, both the reviewers (H.O. and M.M.K.) assessed the quality of RCTs using the Cochrane risk of bias tool for randomized control trials to determine whether there was a low, high, or unclear risk of bias.

### Statistical analysis and outcomes

It was done using the RevMan 5.4.1 software package provided by the Cochrane Collaboration. The meta-analysis presented hair density as continuous data, using a calculated mean and SD of various PRP treatment interventions versus a control. A mean difference with a confidence interval of 95% was used to analyze the data set of hair density and hair diameter, and a *P*-value <0.05 was considered statistically significant. The primary outcome that was assessed and analyzed was hair density (number of hairs/0.65 cm^2^). Studies used global photography and phototrichogram to count the number of hair present in a fixed 0.65 or 1 cm^2^ area of the scalp. Hair density was taken before treatment started and repeated at subsequent visits in both the treatment and control groups. The results at 3 months and 6-month follow-up visits were used to conduct the analysis. All the data extracted was tabulated first on Microsoft Excel and later it was entered into RevMan software where forest plots were generated to evaluate the effect of PRP on hair density. Effect sizes and 95% CIs for the PRP and PRP in combination with other drugs were calculated using a weighted DerSimonianLaird random-effects model^20^. To determine whether any particular study had an impact on the findings or increased heterogeneity, a leave-one-out sensitivity analysis was performed. Egger's regression analysis, funnel diagrams, and Begg’s test are used to statistically analyze the risk of publication bias (*P*>0.05 indicates a lack of publication bias). By using the Cochran Q statistic, heterogeneity was evaluated; 0.1 denotes substantial heterogeneity. Then *I* ^2^ test was used to assess the heterogeneity between the trials, with a scale set at values of 25% for low risk, 25–75% for moderate risk, and greater than 75% for high risk^21^. In all cases, a *P*-value of 0.05 or lower was deemed to be significant.

## Results

**Figure 1:**
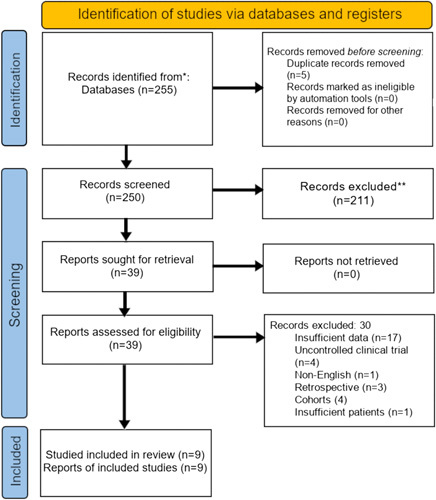
255 records were identified from database searches, and 0 additional articles were identified by other methods. After five duplicates were removed, a screen of 250 articles by title and abstract resulted in 39 articles that were used for full-text review. A full-text review of these 39 articles led to the exclusion of 30 articles for reasons mentioned in the diagram.

Initial literature research with relevant keywords yielded 255 articles. After narrowing the search to 39 studies, 17 were excluded due to insufficient data available, 4 were cohorts, 3 were retrospective/case series, 1 was excluded because it was not published in English and 4 studies were uncontrolled clinical trials. One study was excluded because of having fewer than 10 patients. Therefore, nine RCTs were considered for meta-analysis based on the inclusion/exclusion criteria discussed above. The article selection process has been summarized in Figure 1.

Cumulative data on 230 participants were collected across nine studies. From the studies collected, the participants’ age ranged between 18–65 years and with a minimum follow-up period of 1 month. Five studies were half and half head studies, with one half, injected with PRP or PRP with intervention and the other half with a placebo (normally 0.9% NaCl saline). Three studies gave PRP and control in separate groups. One study injected PRP and a control in selected areas of the same scalp^22^. Patient demographics have been summarized in Table 1.

**Table 1:** Study type and patient demographics

References	Country	Study type	Control type	#treated #control	#males (AGA stage)^*^ #females (AGA stage)	Age (year range)
Gentile et al.,^41^	Italy	RCT, single	Saline (half headed)	23,23	23 (IIa–IV),0	19–63
Alves et al.,^26^	Spain	RCT, double	Saline (half headed)	25,25	12 (II–IV), 13 (I–III)	18–65
Alves et al.,^9^	Spain	RCT, double	Placebo (half headed)	24,24	11 (II–V),13 (I–IIII)	18–65
Gentile et al.,^42^ Group A-PRP	Italy	RCT, double Placebo (half headed)		18,18	18 (II–IV), 0	19–63
Tawfik et al.,^22^	Egypt	RCT, single	saline	30,30	0,30 (I–III)	20–45
Hausauer et al.,^23^	USA	RCT, single	second type treatment	20,20	30 (II–V), 10 (II–III)	18–60
Singh et al.,^25^	India	RCT	Normal saline	20,20	40 (II–V),0	18–60
Dicile et al.,^26^	Turkey	RCT	Placebo	10,15	25 (II–V),0	18 above
Shapiro et al.,^22^	USA	RCT	Saline (half headed)	35,35	18 (III–V),17 (III–V)	18–58

* Alopecia staging for males is by Hamilton-Norwood Scale and for females is by Ludwig Scale. RCT: Randomized Controlled Trial.

Out of the seven studies that reported effects on hair density, all showed an increase after applying PRP or PRP and another intervention from baseline. Seven studies showed statistical significance (*P*<0.05) and only one study reported no difference^23^ when PRP was compared with control.

### Hair density

**Figure 2:**
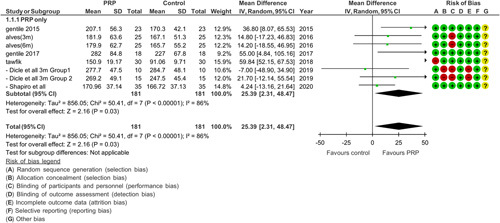
Meta-analysis of hair density data from six randomized controlled trials demonstrates that groups treated with PRP had statistically significant greater hair density than control.

Six RCTs were used to compare the efficacy of PRP with a control (placebo/saline) as demonstrated in Figure 2. A significant increase in hair density (*P*<0.00001, MD=25.39 with 95% CI; *I* ^*2*^: 86%) was noted with PRP only. Heterogeneity decreased (*I* ^2^=18%) when Tawfik *et al.* ^22^ were excluded from the analysis.

**Figure 3:**
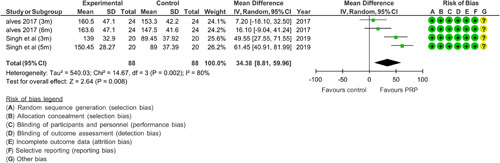
Meta-analysis of hair density data from two randomized controlled trials demonstrates that groups treated with PRP along with another intervention had significantly greater hair density than the control. Alves 2017 used minoxidil and finasteride with PRP. Singh *et al*. used a 5% minoxidil solution along with PRP to treat androgenic alopecia.

Figure 3 shows another comparison between PRP and another intervention vs. control (placebo/saline). There was a significant increase in hair density in the experimental group (*P*=0.002, MD=34.38 with 95% CI) with heterogeneity coming out to be 80%.

**Figure 4:**
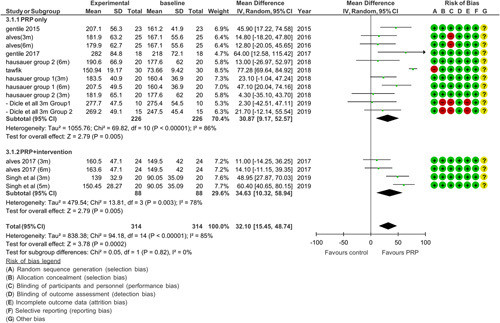
Hair density comparison between PRP and baseline. Subgroup 3.1.1 shows a comparison between PRP and control. Subgroup 3.1.2 shows a comparison between PRP added with intervention and baseline.

Six RCTs were used to demonstrate a comparison between PRP and baseline values. A subgroup analysis was done to compare the efficacy of PRP when given as monotherapy or when combined with another intervention. Results showed that both subgroups had a significant effect on hair density (*P*<0.05) as shown in Figure 4. PRP (monotherapy) had a significant difference in hair growth (*P*<0.00001 MD=36.16 CI 95%). Heterogeneity came out to be 84% but decreased to 11% by doing a sensitivity analysis in which one study^22^ was excluded the possible reason that we identify is that the treatment was applied more frequently and the patients had Egyptian hair type in comparison to studies conducted in the United States clinics^24^.

There was a significant difference (*P*=0.003, MD=34.63 CI 95%) when PRP was used in adjunct to other therapies that were previously used for treating AGA. Heterogeneity was 78% because the study by Singh *et al.* ^25^, has a different method of application from Alves and Grimalt^9^, as it applies intervention and control on two separate sample groups whereas Alves is a half and half head study.

### Bias

Risk of bias was done using the Cochrane risk of bias analysis of the nine RCTs as shown in Figures 2, 3, and 4. Overall, the majority of the studies had a low risk of bias. One of the studies included had a high risk in which there was bias in allocation concealment as it was done by the draw method. Also, there was no mention of any blinding method and 33% of the participants collectively withdrew from the study, thereby making this study have the most bias^26^. In a study conducted by Alves and Grimalt^27^, the difference between the control and PRP intervention was known by the administering physician. All studies mentioned carrying out randomization. One of the studies^22^ carried out randomization using the coin toss method and did not use appropriate software, which increased the risk of bias in the randomization process.

### Adverse effect

Six studies reported adverse effects such as mild local pain, head or scalp sensitivity, pinpoint bleeding, and itchiness just after the procedure as shown in Table 2. There was an immediate reaction such as pain and itching due to a hypersensitivity reaction at the site of injection^23,25^.

**Table 2:** Adverse effects

References	Follow-up	Adverse effects
Gentile et al.,^41^	2 years	NR
Alves et al.,^26^	6 months	Pain
Alves et al.,^9^	6 months	NR
Gentile et al.,^42^ Group A-PRP	6 months	NR
Tawfik et al.,^22^	6 months	Pain, pinpoint bleeding
Hausauer et al.,^23^	6 months	pain, headache, itching
Singh et al.,^25^	5 months	Pain
Dicile et al.,^26^	9 months	pain
Shapiro et al.,^22^	2 years	Pain itching

NR=not reported.

## Discussion

The purpose of this meta-analysis was to analyze and compare the efficacy of the different modalities of treatment for AGA, particularly the use of PRP as a lone treatment and in conjunction with other previous treatments such as minoxidil, finasteride, or micro-needling on hair density, and growth. It can be seen that PRP when given as monotherapy or given in conjunction, has a significant effect on hair density.

Our meta-analysis comprising RCTs provides compelling evidence regarding the efficacy of PRP therapy in increasing hair density when compared both to the baseline measurements and in the control population, which is consistent with the findings reported by the previous studies assessing the correlation^16,17,28^. A subgroup analysis of studies reporting baseline values with PRP alone and with the intervention was performed that yielded similar results on hair density; hence, it affirms that adding an intervention with PRP therapy also shows a significant difference in the hair density.

The advent of PRP therapy has been explored with keen interest as the use of blood products rich in platelets has been indicated for poorly healing scars^29^ and studies have also been conducted on its efficacy on muscle and soft tissue tears, with most showing mixed results. A speedy recovery relative to conservative treatment has been proposed^30,31^. PRP treatment for AGA is suggested to act by various mechanisms. ‘Activated platelets’ release growth factors and cytokines to promote hair growth, which includes platelet-derived growth factor, transforming growth factor-beta, insulin-like growth factor-1, and vascular endothelial growth factor, among others^29^. The treatment promotes cell proliferation and differentiation, angiogenesis, and chemotaxis to improve follicular health and promote regrowth^32^. Activated PRP is hypothesized to prolong the anagen phase, preventing apoptosis and the catagen phase, and also reducing the telogen to the anagen transition period in the hair follicle life cycle^33,34^ hence improving hair diameter and density. However, it is important to note that factors such as PRP preparation and application technique, as well as PRP concentration can greatly affect the outcome of therapy. Over the years minoxidil and finasteride have cemented their place as first-line therapy. These drugs have shown efficacy and tolerability in previous studies by halting of hair loss and significant regrowth of hair. These therapies require life-long continuation to maintain their effect^35^.

Topical preparations of 5% minoxidil, applied directly to the affected scalp, have proven to be extremely efficacious in increasing the anagen phase of the hair cycle and simultaneously shortening the telogen phase. It has also been shown to improve hair follicle size^36^. Minoxidil has a vasodilatory effect and it induces the production of vascular endothelial growth factor to maintain the size and vascularity of dermal papillae that helps sustain the hair follicle. Since the release of various growth factors is also the suggested action of PRP^34^, it is understandable that both minoxidil and PRP have a similar effect on hair growth. Finasteride is a 5 α reductase inhibitor. It inhibits the production of dihydrotestosterone (DHT) and has been a mainstay treatment for AGA and has been shown to reduce serum and scalp DHT by up to 70%^37^. By inhibiting the conversion of testosterone to DHT, finasteride, among other 5 α reductase inhibitors, has been suggested to increase the number of active hair follicles^38^. However, the adverse sexual effects (reduced sperm count, gynecomastia, decreased libido, ejaculatory, and erectile dysfunctions) of finasteride may be a difficult trade-off for many individuals^39^. Further studies need to be conducted on the efficacy of finasteride as an adjunct to PRP therapy.

Other techniques that are gaining prominence in androgenetic alopecia treatment are the use of adipose-derived mesenchymal stem cells, stromal vascular function cells, and human follicle stem cells. These novel methods activate mediators via various biochemical pathways, which result in angiogenesis and tissue regeneration^40^. These stem cell derivatives have been shown to have promising results. However, as they are not yet used commonly in conjunction with PRP, they are beyond the scope of this systematic review and meta-analysis.

### Clinical and research implication

The results imply that while the use of PRP to treat AGA is beneficial, the efficacy of adopting a combined therapy is questionable due to the limited number of data available. Combinations of PRP coupled with finasteride, minoxidil, or micro-needling have all shown a significant increase in hair density, whereas PRP alone also showed a significant increase in hair density. Therefore, it would be prudent to consider PRP a mainstream therapy due to its promising results. Also giving PRP as monotherapy will reduce cost and resources when compared with adjuvant therapy. There is a lack of studies on combination therapies and there is a need of conducting more clinical trials with different settings and populations. There is a need to conduct more prospective and retrospective studies so subgroup analysis could yield better results.

## Limitations

The limitation of this study is that there was lack of access to individual patient data and the analysis was based on the data the authors of the studies deemed relevant. Therefore, we were unable to categorically deny the involvement of other confounding factors in making the results significant. Secondly, there was a lack of studies that evaluated the effect of these interventions on hair diameter.

An unaccounted factor may be the publication bias, as many studies may have not reached print. It is possible that studies that did not yield statistically significant results, did not conform to a particular researcher's beliefs, or were disregarded for any other reason, might have affected the results. There is still unexplained heterogeneity in the meta-analysis that diminishes any conclusions drawn from the study.

Future RCTs need to assess the efficacy of combining PRP with finasteride or minoxidil and determine, which combination is superior. RCTs are also required to confirm if PRP therapy has any significant effect on the hair diameter as compared to other available FDA-approved nonsurgical treatment modalities. Currently, the available literature is insufficient to draw any conclusions. Moreover, additional RCTs will enable researchers to comprehensively assess the outcomes in patients receiving this therapy concerning their age, sex, and grades of AGA.

## Conclusion

PRP therapy is a minimally invasive and inexpensive procedure that has shown promising results compared to the other treatments, with only a few reported adverse effects. The current meta-analysis shows that it could be used as a lone therapy to increase hair density in AGA. It would be a breakthrough in the treatment of AGA if PRP therapy is included in the current treatment protocols for both men and women. Future RCTs need to focus on preparing combinations to assess if it further improves efficacy.

## Ethical approval

Not available.

## Consent

Not available.

## Source of funding

No funding was received for the project.

## Author contribution

K.F.: analysis and review; H.O.: analysis and results; Z.A.: writing the paper (Introduction); M.A.L.M.: writing the paper (method); M.M.K.: writing the paper (method); A.H.K.: writing the paper (Abstract and discussion); A.S.G.: writing the paper (discussion); M.A.K.: writing the paper (discussion); A.J.: writing the paper (introduction); Y.I.M.: interpretation; S.K.: interpretation; A.M.R.: writing the paper and review.

## Conflict of interest

None.

## Research registration unique identifying number (UIN)

Meta-analysis has been registered with Research Registry and the unique identifying number is reviewregistry1546 (https://www.researchregistry.com/browse-the-registry#registryofsystematicreviewsmeta-analyses/).

No amendments were made after the submission of the protocol to the research registry.

## Guarantor

Haziq Ovais.

## Provenance and peer review

Not commissioned, externally peer-reviewed.
